# Assessment of Changes over Time of Lipid Profile, C-Reactive Protein Level and Body Mass Index in Teenagers and Young Adults on Different Diets Belonging to Autism Spectrum Disorder

**DOI:** 10.3390/nu12092594

**Published:** 2020-08-26

**Authors:** Anna Błażewicz, Iwona Szymańska, Aleksander Astel, Agnieszka Stenzel-Bembenek, Wojciech Remington Dolliver, Agata Makarewicz

**Affiliations:** 1 Chair of Chemistry, Department of Analytical Chemistry, Medical University of Lublin, 4A Chodźki Street, 20-093 Lublin, Poland; 2 Faculty of Medicine, Student Research Team, Department of Analytical Chemistry, Medical University of Lublin, 20-093 Lublin, Poland; iwonka.szyms@gmail.com; 3 Department of Biology and Earth Sciences, Pomeranian University in Słupsk, 22a Arciszewskiego Street, 76-200 Słupsk, Poland; aleksander.astel@apsl.edu.pl; 4 Department of Biochemistry and Molecular Biology, Medical University of Lublin, 1 Chodźki Street, 20-093 Lublin, Poland; agnieszka.stenzel-bembenek@umlub.pl; 5 The Division of Pulmonary and Critical Care Medicine, Brigham and Women’s Hospital, 75 Francis Street, Boston, MA 02115, USA; wojciechdolliver@hotmail.com; 6 I Department of Psychiatry, Psychotherapy and Early Intervention, Medical University of Lublin, 1 Gluska Street, 20-439 Lublin, Poland; chemsitry_coordinator@umlub.pl

**Keywords:** autism, lipid profile, C-reactive protein, body mass index, diets

## Abstract

Background: Numerous scientific studies on patients with autism spectrum disorder (ASD) suggest a significant role of inflammation processes or lipid disorders in this spectrum of neurodevelopmental disorders. Unfortunately, there is a lack of assessments of changes over time regarding level of lipids and inflammatory markers in people diagnosed with ASD using different diets. The aim of this study was to evaluate changes in lipid profile, high sensitivity C-reactive protein (hs-CRP) and body mass index (BMI) in individuals diagnosed with ASD and healthy controls. Variables were assessed at two time points (2015/17 and 2017/20) for each subject. Methods: After applying the selection criteria, for the first assessment period, 96 participants were qualified (the group consisted of 59 males with ASD and 37 healthy volunteers, i.e., age-matched control group—CG). The final assessment included 93 participants (57 from ASD group and 36 from CG). Subjects were on low-fat diet (LFD), gluten–casein-free diet (GF–CF) and regular diet (RD), respectively. All members of CG were on regular diet. A fasting lipid profile and hs-CRP level were analyzed. BMI and percentiles were calculated. Eating habits were checked by analyzing data from questionnaires. Principal component analysis (PCA) was used separately for every assessment. The Mann–Whitney U test was used to compare the medians of variables in the scheme of pairwise comparisons between control and ASD groups on different diets for separate assessment, while differences over time between variables were tested by Wilcoxon signed-rank test. Results: Statistically significant differences between BMI, CRP, triglycerides (TG), total cholesterol (TC), high-density lipoprotein (HDL), non-HDL-C and TC/HDL ratio were found in ASD group in comparison to healthy volunteers (increased BMI, CRP and TC/HDL and decreased HDL-C for all types of diets, increased TG in the group of LFD and RD individual and increased non-HDL-C in the group of GF–CF and RD individuals) during the first assessment period. The second assessment over time also showed increased levels of TC, non HDL-C and TC/HDL and decreased level of HDL-C for all ASD individuals regardless of diets used, while BMI and CRP increased only for individuals on LFD and RD. No statistically significant correlations between age of participants and other variables comparing with CG were found. Conclusions: Our studies suggest that targeted, individualized nutritional pattern and periodic screening for lipid and immune disorders would be beneficial for teenagers and adults diagnosed with ASD.

## 1. Introduction

Autism spectrum disorder (ASD) includes several disorders with a wide range of intensity of symptoms and behaviors and of considerable individual variation. A diagnosis of ASD currently includes several conditions that used to be diagnosed separately: Autistic disorder, pervasive developmental disorder not otherwise specified (PDD-NOS) and Asperger syndrome (AS). These conditions are now all called autism spectrum disorder [1,2,3]. Recently, attention has been paid to emphasizing neurodiversity in relation to the term “autism”, which adds additional value to the topic and does not stigmatize people with ASD, who experience many severe problems in everyday life [4]. The etiology of ASD still remains unknown; however it has been suggested that environmental, genetic, social and developmental factors all play a considerable role [5,6,7]. It must be emphasized that the spectrum of disorders is frequently associated with a known medical or genetic condition. There are numerous environmental factors, neurodevelopmental, mental and behavioral disorders accompanying ASD.

Conventional treatment of autism is based on the combination of behavioral therapy and pharmacotherapy. Unfortunately, current pharmacotherapy does not guarantee complete relief of the many, complex symptoms of ASD. Moreover, commonly prescribed medications induce a variety of side effects. Some of these side effects are nutrition-related, for example decreased or increased appetite, vomiting, diarrhea and gastrointestinal irritation [8]. Scientists are undertaking research into the role of nutrition, dietary supplements and therapies of people with ASD due to the demonstrated significant influence of nutrition on both brain function and its biochemistry. There is a growing body of evidence on nutrient deficiencies—the relatedness of function of important neurotransmitters (dopamine, serotonin, acetylcholine and γ-aminobutyric acid) in the brain and many dietary components are currently suspected to be associated with alterations in behavior and cognition [9,10]. Particular attention is being paid to deficiencies of vitamin D, K, pantothenic acid, calcium and several bio elements including iron, iodine and selenium [7,11,12,13,14]. A high incidence of metabolic imbalances in autism, e.g., decrease in the methylation capacity (folate cycle, transmethylation and transsulfuration biochemical pathways) and resulting oxidative stress, as well as the significance of metabolic activity of the microbiome in autism are highlighted in literature [10,15,16]. Numerous studies on ASD emphasize the importance of gut-associated immune responses and suggest that diet is associated with the regulation of inflammation [17,18]. It is suggested that inflammatory markers and lipids may play an important role in the pathophysiology of neuropsychiatric disorders, including autism [19,20]. Nonetheless, there are no mandatory periodic screening tests of lipid profile in individuals diagnosed with autism.

Considering the evidence listed above, it is highly likely people with autism could benefit from a properly selected diet therapy. Although eliminating certain foods or food items from the diet (e.g., casein and gluten free) is increasing popular treatment approach, there is a lack of consistent and good quality scientific evidence to support their recommendation as a treatment method. To date, most studies discussing dietary approach to the management of that complex spectrum of disorders focus not only on the possible benefits of various nutritional interventions (via supplementation of deficient components of a diet, e.g., vitamins, minerals, probiotics, omega-3 fatty acid, amino acids), but also emphasize insufficient evidence of its effectiveness and a lack of statistical power for evidence-based treatment recommendations [21,22,23,24,25].

Current nutritional studies in the ASD population mainly concern children, and dietary intervention studies on patients with autism are very rarely performed in adulthood. It was reported that inadequate intake of some micronutrients are highly significant especially in individuals at older age, when the risk of developing diet-related diseases such as obesity and cardiovascular disease is much higher than in childhood [18,26]. However, research on nutrition, diets and their effects on the older group of people with ASD is still rare.

Taking into consideration the lack of repeated studies over time in a specific population of young adults and teenagers with autism in the aspect of combined dietetic and biochemical analyses, we have adopted a research strategy that allows us to observe the same groups repeatedly, at two time points, from a dual—dietary and biochemical perspective. Our first intention was to check whether there are any differences in lipid profile or CRP levels in the population of people with ASD, compared to fully healthy individuals at the same age. Another one was to assess fluctuations of that difference over time, taking into account the type of used diet. According to our knowledge there are no such studies so far. The main aims of this study were to assess the changes over time of lipid profile, CRP level and BMI in individuals diagnosed with autism and healthy controls and to assess the factors that may affect these variations, mainly type of diet.

## 2. Materials and Methods

### 2.1. Subjects and Variables

Studies were done on a carefully and thoroughly chosen population diagnosed with ASD. Patients were recruited through local support groups (mainly parents) or were referred by specialized clinicians and therapists. Informed consent of parents of children or currently adult subjects was obtained. The study was conducted in accordance with the Declaration of Helsinki and the protocol was approved by the Bioethics Committee of the Medical University of Lublin, Al. Racławickie 1, 20-059 Lublin, Poland (number of the approval: KE-0254/12/2014).

Data were collected and analyzed in two periods, namely between 2015/2017 (first assessment) and 2017/2020 (second assessment) from ASD patients and healthy controls matched for age, who were unrelated to the subjects. Although our research project did not exclude any of the sexes, only boys and young men were qualified for the study because they met the selection criteria listed below.

The diagnosis of ASD was performed based on the criteria for autistic disorder as defined in the Diagnostic and Statistical Manual of Mental Disorders, Fifth Edition (DSMV), Autism diagnostic interview revised (ADI-R) and autism rating scale (CARS) [1].

For the purpose of the present study, we considered only the diagnosis and not disorder severity. Subjects with familial hypercholesterolemia and those having any acute or chronic illness (except psychiatric diagnose of ASD) were excluded from this study. The presence of any pharmacotherapy (including cholesterol-lowering drugs or medications with known effects on lipid levels), alcohol consumption and active smoking during and one month before the tests were the basis for removal from the study. The participants took part in a parallel project on the analysis of micro- and macroelements, therefore the use of mineral and vitamin preparations, additional dietary supplements was a contraindication to participate in this study. The place of residence, time from the diagnosis or other socioeconomic data are not analyzed in the present work.

After applying the selection criteria, for the first assessment period, 96 participants were qualified (the group consisted of 59 males with ASD and 37 healthy volunteers, i.e., control group—CG). The final, second assessment included 93 participants (57 from ASD group and 36 from CG group); three participants were excluded from the study, due to the use of dietary supplements (1) and the use of psychotropic medication (2).

The data on age of patients and the healthy volunteers participating in the two periods of the study (i.e., during the first and second assessment periods) are included in Table 1. The body mass index (BMI) was calculated in the following way: weight (in kg)/(height in m)^2^. Normal weight for the younger group was defined by a BMI < 85th percentile, overweight by a BMI between the 85th and 95th percentile and obesity by a BMI ≥ 95th percentile. For children and youth, BMI is age and gender specific and this was considered when interpreting their BMI. Percentile charts were applied, and the thresholds were given below Table 1, respectively, because our patients both younger than 18 years and older than 18 y participated during first period of tests. In the second period of measurements all the patients and control subjects were already adults.

Lipid profile (blood triglycerides—TG, total cholesterol—TC, high density cholesterol—HDL-C, non-high density cholesterol—non-HDL-C, ratio of total cholesterol to high density cholesterol—TC/HDL), high-sensitivity C-reactive protein—(hs-CRP), as well as dietary information were observed between: 2015/2017 (first assessment), and again between 2017/2020 (second assessment) (Table 1). Biochemical tests were carried out by accredited analytical and diagnostic laboratories in Masovian and Lublin (central and southeast Poland) regions in Poland. Laboratory blood tests were performed for periodic prophylaxis purposes.

The eating habits, types of diets were checked by analyzing data from questionnaires which were designed for self-administration by participants with the assistance of their parent or caregiver. Taking into consideration that possible differences in food choices exist due to different age and severity of the disorder’s symptoms the questionnaires contained a complete list with pictures of the foods that were consumed by the population under study. A review of the diet history, frequency of food avoidance episodes, physical examination, growth and anthropometric measurements, were collected before performing laboratory tests. A verification of the sources of specific dietary recommendations was not the intention of this study. We received information from the parents of patients, who were mostly motivated by a specific diet choice because of the suggestion of the attending physician, therapist, physiotherapist or their own/parents’ beliefs about the effectiveness of the chosen diet.

In Table 1 we categorized the study participants according to the types of diets used into the following groups: RD—i.e., the regular diet—a typical diet assumed to meet all the requirements of the principles of rational nutrition for healthy people that does not include any dietary restrictions; LFD—a low-fat diet in which no more than 25–30% percent of calories come from fat and where animal fat, simple sugars and cholesterol intake are reduced; GF–CF—gluten and casein-free diet—a strict elimination diet, where all foods containing gluten and casein are removed from the daily food intake. It must be emphasized here that according to parents’ reports no tests confirming any allergy were conducted ever before. Cases of combined diets, e.g., GF–CF + LFD were eliminated for purposes of the present study. None of the study participants confirmed a diet change during the periods of our studies. The control group members were on a regular diet during entire period of tests. The authors of the project did not require patients to follow any diet.

### 2.2. Statistics

For overall exploration of the data sets including variables: BMI, age, HDL-C, CRP, TG, TC, non-HDL-C and TC/HDL principal component analysis (PCA) was used separately for every assessment. It produces a low-dimensional representation of a data set, finds a sequence of linear combinations of the variables that have maximal variance and serves as an efficient tool for data visualization. In the case of current research, it was necessarily applied as exploratory step to reveal if ASD individuals on specific diets could be somehow spread in multidimensional space according to direction of the most contributing variables and if ASD individuals create groups somehow separated from CG. As an extraordinarily efficient tool of exploratory analysis, it usually justifies an application of consecutive testing, as was also in this case. Prior its use the interest of the implementation of the PCA on a data set was double checked using analysis of Spearman’s correlation matrix as well as Bartlett’s sphericity test. However, the PCA itself was calculated in its default mode using Pearson’s correlation coefficient instead of its derivation with rank-based correlation matrix since the goal of its use was to glance on multidimensional space and none of any further models using PCs were not calculated.

**Figure 1:**
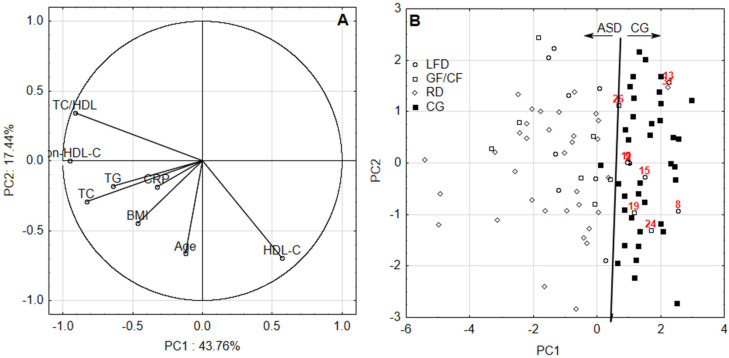
(**A**) The first principal component (PC1) and the second principal component (PC2) loadings and (**B**) scores for first data set; ASD—group, CG—control group, LFD—low-fat diet, GF–CF—gluten-free/casein-free diet, RD—regular diet.

**Figure 2:**
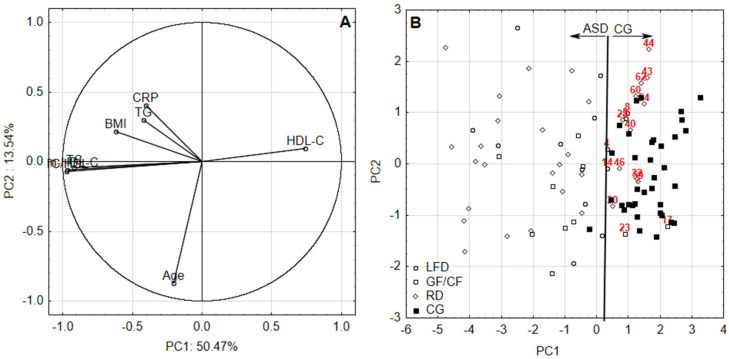
(**A**) The first principal component (PC1) and the second principal component (PC2) and (**B**) scores for second data set. ASD—group, CG—control group, LFD—low-fat diet, GF–CF—gluten-free/casein-free diet, RD—regular diet.

In this study, Spearman’s correlation coefficient (*p* = 0.001) had to be applied for all combinations of variables since their distribution was far from Gaussian, which was checked in advance by the use of Shapiro–Wilk test (*p* = 0.05). Since plenty of statistically significant correlations were found, the next step of checking was commenced. Bartlett’s test compared the observed correlation matrix to the identity matrix. In other words, it checked if there was a certain redundancy between the variables that can be summarized with a few number of principal components. If the variables were perfectly correlated, only one factor was sufficient. If they were orthogonal, as many factors as variables were needed. In this last case, the correlation matrix was the same as the identity matrix. In all assessments, the chi-squared test statistics were approximately tenfold higher than the critical value, checked at a significance level *p* = 0.05 and hence, all data sets were suitable for PCA. As mentioned above an application of PCA justified selection of appropriate further tests since its visualization ability, factor scores plot in particular, became extremely beneficial in the case of current research. With four unmatched groups of individuals (ASD on RD, LFD, GF–CF and CG on RD) for every period of assessment in the mode of between-groups comparison the original tests selection should involve one-way analysis of variance by ranks following-up by the post hoc multiple comparisons test. However, PCA score plot revealed overlapping patterns of ASD individuals in specific diets (presented further as Figure 1B and Figure 2B) suggesting none of differences among ranked values of their blood characteristics. The lack of any significant differences in every combination of comparisons for ASD individuals was confirmed during the preparatory step and this is why results concerning the application of one-way analysis of variance by ranks following-up by the post hoc multiple comparisons test was skipped in the study. The only statistical differences found concerned comparison between ASD individuals on specific diets with CG on RD; this is why tests suitable for comparisons in the mode of two unmatched or matched groups were chosen.

Due to the non-normal distribution of values for majority of variables the Mann–Whitney U (*p* = 0.05) and Wilcoxon signed-rank (*p* = 0.05) tests were selected as the best choice for between-group and within-group comparisons, respectively. Since the Mann–Whitney test is a test of both location of the dominant values and the shape of data distribution it was applied to detect differences in medians of variables in the scheme of pairwise comparisons between CG (RD) and ASD on other diets (RD, LFD, GF–CF) for separate assessment, while within-group differences over time between variables were tested by Wilcoxon signed-rank test. It was taken into consideration that differences in variables median are often accompanied by other differences in spread and shape of variables distribution and in large samples the Mann–Whitney U test can detect differences in spread even when the medians are very similar. However, in the case of this particular study due to the limited number of individuals in each group the idea of analyzing and testing the spread and shape of variables distribution was of minor importance and hence the discussion concerning medians has become a priority. All calculations were performed by the use of the software package TIBCO Statistica 13.3 (Tibco, Inc., Palo Alto, CA, USA).

## 3. Results

A summary of patient and control data and their comparison is presented in Table 1.

As mentioned above, due to the lack of Gaussian data distribution, Spearman’s correlation coefficient matrix was calculated between investigated parameters according to consecutive assessments (Table 2).

Highly significant (*p* = 0.001) correlation coefficients between BMI, CRP and lipid profile proved the application of PCA was justified. According to the PCA, two factors with eigen values >1 were obtained for all assessments, accounting for 61.20% and 64.01% of the total variance for first and second data set, respectively. The relation between identified components and variables as well as patients and healthy volunteers according to their diets was identified by the visualization of factor loadings and factor scores in the combination of two principal components (Figure 1 and Figure 2). Convergent signs of factor scores and correlations between variables contributed to a factor indicates directly proportional relation—the higher the positive value of the factor score, the higher the impact (in terms of value) of positively correlated variable with the factor on the sample. Moreover, conversely, the higher the positive value of the factor score, the lower the impact of the negatively correlated variable with the factor on the sample.

The Spearman’s correlation coefficient (*p* = 0.05) matrix between age, BMI, CRP and lipid profile determined in investigated patients and healthy volunteers according to first and second period of the assessment and type of diet is summarized in Table 3.

The differences between variables over time were tested by Wilcoxon signed-rank test (Table 4).

## 4. Discussion

The importance of cholesterol in the body is the subject of many scientific studies. It is essential for neuroactive steroid production and growth of myelin membranes. It also enables normal embryonic and fetal development. Cholesterol modulates the oxytocin receptor, ligand activity and G-protein coupling of the serotonin 1A receptor [27]. Unfortunately, the full role of cholesterol in the context of ASD is still unknown. The results of various, mainly cross-sectional studies indicate the presence of dyslipidemia in boys with autism and suggest an association between lipid metabolism and ASD [28,29]. Other studies indicate a higher prevalence of hypertriglyceridemia and obesity in individuals with autism when compared to the general population, independently from lifestyle, obsessive–compulsive behavior and social anxiety [29]. Since each lipid panel varies depending on the existing risk factors (age, family history, existing conditions like diabetes, etc.), we carefully and thoroughly selected patients for the study (diabetes cases were not considered). The comparison of our ASD group with the control group (i.e., age-matched healthy participants) revealed significant disparities in several tested parameters (Table 1).

Findings of the first period of our study (i.e., first assessment period) revealed statistically significant differences between BMI and levels of CRP, triglycerides (TG), total cholesterol (TC), high-density lipoprotein (HDL), non-HDL-C and TC/HDL found in ASD group in comparison to healthy volunteers. Patients diagnosed with ASD had increased BMI and levels of CRP and TC/HDL and decreased HDL-C regardless of the diet used. For the participants of ASD group being on LFD or RD diet increased TG levels were observed and increased non-HDL-C in the group of GF–CF and RD individuals, respectively. It is generally accepted that non-HDL levels are a better predictor than the LDL alone because they include other apolipoprotein B (Apo B) containing particles like very low-density lipoprotein (VLDL), intermediate density lipoprotein (IDL) and lipoprotein (Lpa).

The second assessment over time (after approximately 3–5 years from the first measurement) on the same groups of participants showed increased levels of TC, non HDL-C and TC/HDL and decreased level of HDL-C for all ASD individuals regardless of diets’ types. The ASD individuals on LFD and RD increased BMI and CRP levels were found, whereas statistically similar BMI and CRP values were observed for GF–CF group. No statistical significance of age for any group at any of the study periods was found.

Without taking into consideration the type of diet Spearman’s correlation analysis for healthy group and patients diagnosed with ASD confirmed that TG levels were notably correlated with BMI in both periods of the study, similarly to the total cholesterol and non-HDL-C. The same significant correlation, regardless of the measurement period, was observed between TC, non-HDL-C and TC/HDLC ratio and CRP level, TC, non-HDL-C, TC/HDL and TG level and also between TC and non-HDL-C and TC/HDL ratio. Non-HDL-C and TC/HDL ratio significantly correlated with HDL-C (Table 2).

Adding type of diet used to the next Spearman’s correlation analysis for individuals on low-fat diet it turned out that BMI correlated significantly with total cholesterol and non-HDL fraction of cholesterol only in the second period of the measurements. The other significant correlations for LFD group referred to TC with non-HDL-C and TC-with-TC/HDL ratio, HDL-C with TC/HDL ratio and non-HDL-C with TC/HDL ratio for both periods of measurements. HDL-C correlated significantly also with non-HDL-C, but it was indicated only during the second assessment. Interesting results have been found in group of ASD patients on GF–CF diet. During the first assessment period CRP level negatively correlated with TC and non-HDL-C, whereas positively with HDL-C level. HDL-C level negatively correlated with non-HDL-C and TC/HDL-C ratio in both periods of measurements. Total cholesterol and non- HDL-C correlated with BMI only for the elderly patients on GF–CF diet. The analysis for the group of ASD patients on a regular diet revealed that TC, non-HDL-C and TC/HDL ratio were significantly correlated with age, but only for the second period of measurements. The same situation referred to the correlation of BMI with TG, TC, non-HDL-C, TC/HDL ratio and TC with TG and HDL-C. The second assessment indicated that HDL-C negatively correlated with non-HDL-C. As Table 3 shows for the healthy group being on regular diet significant correlations were found between BMI, TG, TC, HDL, non-HDL-C and age only for the first period of tests and interestingly, BMI correlated with non-HDL-C and TC/HDL ratio only in the later period (Table 3).

PCA analysis suggests the following: the first component is able to discriminate between ASD and CG with the highest contribution of HDL-C (positive loading) and non-HDL-C, TC/HDL, TC and TG (negative loadings) (Figure 1 and Figure 2). Increased values of TC/HDL, non-HDL-C, TC, CRP, BMI and TC were observed for ASD patients. Among them there were no striking differences depending on the diet. It was noticed, however, that patients on RD diet present with increased CRP, BMI and TG. On the other hand, patients following GF–CF diet have higher TC/HDL and non-HDL-C. It turned out that in the first considered period of ASD patients, TC/HDL, non-HDL-C, TG and TC levels were increased. It seems like the LFD diet increases the resemblance of the aforementioned values of ASD patients to the values noted in the control group. In turn, RD diet diminishes this resemblance to the greatest extent. Therefore, a suggestion to lower dietary fat or change the diet to LFD in ASD patients arises. It appears that RD is least beneficial for these patients at that age.

In the next assessment period, among older patients with ASD, increased values of TC, non-HDL-C, TC/HDL, TG and CRP were noted. It appears that LFD brings the measured values of ASD participants closer to those of the control group, while RD brings them apart to the greatest extent. The results deliver some clues that RD is least favorable for ASD patients. We are aware, though, that this finding requires confirmation with a bigger group of patients. CRP was the most significant studied parameter in this developmental period. Increased CRP levels serve as a nonspecific indicator for the presence and extent of inflammation processes, rather than as an indicator of a specific pathology. C-reactive protein level is normally found at concentrations of less than 10 mg/L in the blood. It is an acute-phase protein produced mainly in the liver by hepatocytes as an early and nonspecific response to different abnormalities in human body. CRP serum level may rapidly increase as a consequence of tissue damage or necrosis; infection (viral, bacterial); parasitic invasion; inflammation; aging or malignant neoplasia. The highest activation of its synthesis is caused by proinflammatory factors such as interleukin-1 (IL-1) and interleukin-6 (IL-6); both released from mononuclear phagocytic cells as stimulators of the synthesis of the majority acute phase reactants. Serum proteomic analysis studies have identified sex-specific differences in lipid metabolism and inflammatory profiles in adults diagnosed with Asperger syndrome [30]. The studies of Steeb et al. [30] revealed that adult males with Asperger syndrome belonging to ASD had alterations in several serum proteins (i.e., TNF-alpha, TF, IL-16, IL-12p70, CTGF, PAP, EPO) involved in the signaling of inflammation. Immunological and inflammatory mechanisms may play an important role in the pathophysiology of some psychotic disorders like schizophrenia and autism [31,32]. Diagnostic differentiation of autism and schizophrenia is usually unproblematic despite some mutual features. Autism criteria include qualitative abnormalities encompassing mutual social interactions, i.e., lack of social-emotional reciprocity, limited development of peer relationship, failure to make eye contact, facial expression, body posture and gestures poorly related to the social interactions. There is also lack of spontaneity in sharing emotions, interests or needs. Generally speaking, schizophrenia appears as a failure to communicate with the outside world, concentration on internal, frequently aberrant experiences. As stated in the introduction, a plethora of coexistent disorders and problems (e.g., obsessive-compulsive disorder, affective disorders, schizophrenia, eating disorders) make it more challenging to diagnose the discussed disorders, especially in adults [33].

Recently, studies increasingly indicate a strong inflammatory state associated with autism [34]. Neuroinflammation and neuroimmune abnormalities have now been established in ASD as key factors in its development and maintenance [35]. Studies suggest that inflammation during pregnancy may be considered as epidemiological and preclinical evidence for a common pathway by which gestational elevation of CRP can cause subsequent immune activation in the new growing organism. Increasing maternal CRP levels were significantly associated with autism in offspring [36]. Maternal immune activation induces the release proinflammatory cytokines, including IL-6, which can lead to the down regulation of placental growth hormone with subsequent effects on fetal brain development—reducing the number of primary dendrites and nodes [37,38]. Epidemiologic studies have also indicated other immune risk factors during early gestation, such as significantly increased levels of interferon-γ (IFN-γ), interleukin-4 (IL-4), interleukin-5 (IL-5), tumor necrosis factor-α (TNF-α), tumor necrosis factor-β (TNF-β), increased interleukin-10 (IL-10) and increased amniotic fluid levels of monocyte chemotactic protein-1 (MCP-1) in autism spectrum disorders [39,40]. Although different immune and inflammatory alterations in the brain and blood are documented in patients with psychiatric disorders, the mechanism by which abnormal inflammatory cytokine levels affect the development of ASD is unclear. A hypothesis for a final pathway involving stress-induced activation of the inflammatory pathway, microglial activation and the TRYCATs pathway has been suggested [40,41]. According to this hypothesis, cytokine-IL-6 can cause persistent activation of brain indoleamine 2,3-dioxygenase with increasing levels of products of tryptophan catabolism (TRYCATs) such as kynurenine, kynurenic acid and quinolinic acid which results increased NMDA (N-methyl-D-aspartate) antagonism, dopaminergic and serotoninergic dysfunction, excitotoxicity and oxidative stress.

The higher CRP levels and higher plasma lipid levels in ASD compared with levels of healthy group observed in the present study suggest that the population of participants diagnosed with autism spectrum disorder may be exposed to subacute infections and/or inflammation. Dyslipidemia is also associated with increased severity of psychotic syndromes, which are sometimes observed in ASD. It is known that patients with ASD very often manifest also depressive symptoms, and some studies on major depression revealed decreased levels of HDL, increased level of LDL and an increased LDL/HDL ratio [42,43]. Although we did not analyze symptoms of depression in the present study our findings (e.g., decreased HDL level, increased non-HDL-C, TC, TC/HDL in ASD group) clearly indicate the presence of lipid imbalance in ASD. It is known that in depression, proteins involved in the signaling of inflammation are increased. Perhaps it is the future detailed analysis of the symptoms of the disorder that will help to reveal the relationship between increased inflammation and the disturbances of lipid levels in our patients.

The differences over time between blood parameters according to the type of diet for our ASD patients and healthy volunteers (as shown in Table 4) are in some degree surprising. For instance, some increase in median values of TG, TC, non-HDL-C and TC/HDL for participants with diagnosed ASD being of LFD is observed. Omitting TG similar increase was observed for patients being on GF–CF. However, in both cases observed differences could not reach statistical significance, probably due to sample size. Although, these differences were insignificant, they may suggest that perhaps nonnutritional factors have a much greater impact on lipid profile and metabolism in ASD than noted commonly in healthy people. In case of ASD individuals being on RD some significant decrease of TG and HDL-C values was observed over time. The highest number of differences between parameters associated with lipid profile (CRP, TG, TC and HDL-C) over time was observed in the group of healthy volunteers.

Throughout the entire period of our study, the subgroup of ASD patients following the regular diet had statistically significant increased BMI than the healthy individuals following a regular diet. Determining variable values of median for the parameters measured in blood and taking into account a diet type in subsequent measuring sessions, it was observed that BMI stabilizes on the upper limit for a healthy person. Normalization was noted in each group of the assessed patients. During consecutive years, BMI remained on the similar level, regardless of the type of diet used. It is known that systemic and low-grade chronic inflammation is associated with central obesity and the diet may play a significant role in the regulation of that process. Obese adipose tissue releases proinflammatory adipokines and cytokines such as leptin, adiponectin, resistin, TNF-α, IL-1 and IL-6, CRP and plasminogen activator inhibitor-1 (PAI-1) which subsequently promotes other proinflammatory pathways and cells for action, stimulates oxidative stress and thrombopoiesis. Greater adiposity leads to a higher CRP level [44]. Some meta-analysis studies also indicate links between depression, psychotic diseases and lipids abnormalities connected to inflammatory processes and immune system dysfunction [45]. Thus, the search for a link between inflammation, fat metabolism disorders, obesity and nutrition in patients with ASD is justified. Because no studies were conducted on the consequences of any restrictive or regular diet for the lipid profile and CRP level in adult ASD population, it is currently impossible to compare our results with other countries.

### Advantages, Limitations and Future Challenges of the Study

The main advantages of our study are its research problem and its design (two periods of measurements on the same group of people according to identical criteria). During the data analysis, the dependent variables were measured at two time points for each subject, over a relatively long period of time (starting 2015–2017 and completing between 2017–2020). The inclusion and exclusion criteria for being in the study were prespecified and applied uniformly to all participants. The study includes a control group matched for age and gender.

We have not yet described the intensity of the symptoms presented by the patients and their association with the type of the diet. Dietary composition and product quality are not included in this work. These data will be analyzed in future studies. The next step, possibly helpful in explaining observed relationships, would be analyzing the influence of physical activity on biochemical parameters we measured. Numerous patients with autism do not only suffer from malabsorption, obsessive or selective eating, but also motor coordination disorders and physical activity aversion. This can notably affect their lipid profile as well as determine dietary preferences. In addition, it is important to take into consideration that observed differences between ASD and healthy groups in some degree may be due to patient’s ongoing development and hormonal changes.

In recent years, the use of various restricted diets has gained popularity, however selective diets in certain patients can lead to the development of various dietary deficiency states (e.g., vitamins and/or micronutrients) [46]. This can play a crucial role in metabolic disturbances associated with autism [46,47,48]. Obesity and excess weight are typically the first stimuli to encourage dietary interventions. Among patients with this disorder, there are known cases of excessive consumption of dairy products or extremely diverse behaviors, such as refusing any type of dairy. Proofs of efficacy of various dietary plans are not always clear, but the possible benefits of a comprehensive nutritional and dietary interventions are described [48,49].

Further trials are required to explore long-term effects of different diets on symptoms and nutritional status in ASD patients. It seems to be important to check whether processed foods or home-made products have an impact on the results. Our study proves, that altering a dietary plan, should not be permanent. It should be adjusted according to results of tested biochemical parameters.

## 5. Conclusions

In autism spectrum disorders, a well-balanced diet (which guarantees the body is provided with the right amount of nutrients necessary to meet its nutritional needs) introduced on a case-by-case basis should be an integral part of treatment and needs to be preceded by laboratory tests. Obtained results from two study periods suggest the presence of lipid-profile disturbances, especially for older ASD individuals. This may be a suggestion for doctors to test the level of lipids more often in patients with autism. Our findings do not confirm the need to follow one specific dietary plan. It is crucial to provide patients with dietician support since every alteration in the previously accepted nutrition scheme may provoke high stress reaction. Our studies confirm that targeted, individualized nutritional pattern and periodic screening for lipid and immune disorders would be beneficial for teenagers and adults diagnosed with ASD.

## Figures and Tables

**Table 1:** Studied variables and their comparison for two assessment periods (IQR—interquartile range; ATT—above threshold teenagers; ATA—above threshold adults).

2015–2017 TESTS									
	Diet	*n*	Mean	Median	Min	Max	IQR	ATT	ATA
Age **	LFD	14 (ASD)	16.6	16.0 (*p* = 0.092)	15.0	20.0	2.0		
BMI			23.9	24.0 * (p = 0.044)	20.0	28.0	3.0	7.1%	
CRP			8.6	6.9 * (p < 0.001)	1.2	24.6	9.9	42.9%	
TG			100.9	99.4 * (p = 0.003)	68.3	129.2	17.3	64.3%	
TC			176.9	175.2 (*p* = 0.154)	125.8	202.3	32.9	50.0%	
HDL-C			52.2	50.5 * (p = 0.029)	36.4	76.8	14.6	28.6%	
non-HDL-C			124.7	123.2 (*p* = 0.078)	82.2	161.0	41.4	50.0%	7.1%
TC/HDL			3.5	3.4 * (p = 0.017)	2.2	5.0	1.2	28.6%	
Age	GF–CF	10 (ASD)	17.8	18.0 (*p* = 0.092)	15.0	20.0	3.0		
BMI			24.1	24.5 * (p = 0.031)	20.0	27.0	2.0		
CRP			5.9	5.6 * (p = 0.002)	1.5	11.8	6.9	20.0%	
TG			104.9	100.1 (*p* = 0.088)	54.9	171.3	41.4	20.0%	20.0%
TC			185.6	184.7 * (p = 0.047)	158.9	216.2	31.6	40.0%	10.0%
HDL-C			49.8	49.5 * (p = 0.167)	36.5	66.5	5.7	30.0%	
non-HDL-C			135.7	129.5 * (p = 0.008)	103.3	175.8	48.3	40.0%	30.0%
TC/HDL			3.9	3.6 * (p = 0.007)	2.6	5.5	1.6	30.0%	
Age	RD	35 (ASD)	17.3	17.0 (*p* = 0.419)	15.0	21.0	3.0		
BMI			25.1	25.0 * (p < 0.001)	20.0	31.0	3.0	14.3%	
CRP			10.9	9.5 * (p < 0.001)	1.5	32.4	14.3	48.6%	
TG			140.8	147.5 * (p < 0.001)	65.5	205.3	70.6	48.6%	22.9%
TC			198.2	199.7 * (p < 0.001)	148.7	260.7	35.5	57.1%	14.3%
HDL-C			49.6	46.9 * (p < 0.001)	35.6	66.9	12.4	37.1%	
non-HDL-C			148.6	146.0 * (p < 0.001)	95.5	213.8	38.1	45.7%	14.3%
TC/HDL			4.1	4.1 * (p < 0.001)	2.8	6.4	1.4	54.3%	
Age	RD	37 (CG)	17.6	18.0	15.0	20.0	3.0		
BMI			22.4	22.0	20.0	25.0	5.0		
CRP			2.0	1.5	0.0	9.1	2.4		
TG			83.9	81.7	45.6	124.3	27.0		
TC			170.6	167.8	128.7	199.8	15.3		
HDL-C			58.7	56.8	46.3	87.6	16.1		
non-HDL-C			111.9	111.9	80.0	129.8	15.4		
TC/HDL			3.0	2.9	2.2	3.7	0.6		
**2017–2020 TESTS**									
	Diet	*n*	Mean	Median	Min	Max	IQR	ATT	ATA
Age	LFD	14 (ASD)	19.6	19.0 (*p* = 0.792)	18.0	23.0	2.0	
BMI			24.4	24.5 * (p = 0.029)	22.1	26.0	1.0	7.1%	
CRP			10.5	8.7 * (p < 0.001)	1.5	32.8	10.0	50.0%	
TG			118.2	117.3 (*p* = 0.128)	80.7	168.2	47.2	7.1%	21.4%
TC			196.2	189.7 * (p < 0.001)	168.9	256.8	18.9	21.4%	14.3%
HDL-C			49.5	48.8 * (p = 0.021)	39.8	60.5	9.2	14.3%	
non-HDL-C			146.7	138.3 * (p < 0.001)	116.3	211.8	24.1	21.4%	64.3%
TC/HDL			4.0	3.9 * (p < 0.001)	2.9	5.7	1.1	50.0%	
Age	GF–CF	10 (ASD)	20.8	21.0 (*p* = 0.075)	18.0	23.0	3.0		
BMI			23.7	24.5 (*p* = 0.215)	20.0	26.0	3.0		
CRP			6.1	4.5 (*p* = 0.057)	0.5	12.5	8.4	40.0%	
TG			99.5	90.5 (*p* = 0.464)	71.3	184.8	19.7	10.0%	10.0%
TC			192.7	197.3 * (p < 0.001)	160.8	211.0	9.8	10.0%	20.0%
HDL-C			45.0	44.7 * (p < 0.001)	36.6	60.2	8.9	60.0%	
non-HDL-C			147.7	152.0 * (p < 0.001)	115.6	164.5	13.1	10.0%	70.0%
TC/HDL			4.4	4.4 * (p < 0.001)	2.9	5.5	0.9	80.0%	
Age	RD	33 (ASD)	19.5	20.0 (*p* = 0.510)	17.0	23.0	3.0		
BMI			25.9	*25.0 (p < 0.001)*	21.0	38.0	4.0	6.1%	
CRP			9.8	*9.5 (p < 0.001)*	2.4	28.2	8.4	45.5%	
TG			110.0	101.3 (*p* = 0.241)	67.2	165.4	41.4	12.1%	12.1%
TC			198.8	*198.7 (p < 0.001)*	156.9	260.8	54.7	12.1%	30.3%
HDL-C			46.3	*44.2 (p < 0.001)*	36.9	60.6	8.5	54.5%	
non-HDL-C			152.5	*153.6 (p < 0.001)*	96.7	216.4	60.7	15.2%	57.6%
TC/HDL			4.4	*4.4 (p < 0.001)*	2.6	6.1	2.1	60.6%	
Age	RD	36 (CG)	19.7	20.0	17.0	22.0	3.0		
BMI			23.2	24.0	20.0	26.0	2.9		
CRP			2.9	2.6	0.1	8.6	3.8		
TG			100.1	99.3	56.8	145.5	27.0		
TC			164.5	165.7	130.6	190.9	12.4		
HDL-C			53.9	55.9	45.2	65.5	9.7		
non-HDL-C			110.6	111.4	81.1	141.0	17.0		
TC/HDL			3.1	3.1	2.4	3.8	0.5		

Note: * italic represents significant differences between median value of the given parameter in ASD and CG (significance level in brackets); ** the threshold refers to accepted standards; the units and cutoff values are as follows: age (years), body mass index (BMI) (>85th percentile for age and gender and above 24.9, for first and second assessment, respectively), C-reactive protein (CRP) (>10 mg/L), triglycerides (TG) (>90 mg/dL and >150 mg/dL—for first and second assessment), total cholesterol (TC) (>170 mg/dL and >200 mg/dL for first and second assessment), high density cholesterol (HDL-C) (<45 mg/dL and <40 mg/dL), HDL-C (<45 mg/dL), non-HDL-C (>120 mg/dL and >130 mg/dL—for first and second assessment period), TC/HDL (>4); n-number of participants; LFD-low fat diet; GF-CF- gluten free-casein free diet; RD-regular diet.

**Table 2:** Spearman correlation coefficient matrix between age, BMI, CRP and lipid profile determined in investigated patients and healthy volunteers according to first and second assessment period.

	Mean	SD	Age	BMI	CRP	TG	TC	HDL-C	Non-HDL-C	TC/HDL
Age	17.4 *	1.7	1.00							
	19.7 **	1.7	1.00							
BMI	23.8	2.6	0.17	1.00						
	24.4	2.6	0.04	1.00						
CRP	6.6	7.2	−0.07	0.19	1.00					
	6.8	6.5	−0.03	0.19	1.00					
TG	109.3	38.5	0.06	*0.47 (p = 0.001)*	*0.32 (p = 0.01)*	1.00				
	106.3	27.6	−0.02	*0.21 (p = 0.05)*	0.05	1.00				
TC	183.1	24.2	0.25	*0.28 (p = 0.01)*	*0.26 (p = 0.05)*	*0.32 (p = 0.01)*	1.00			
	184.5	27.4	*0.23 (p = 0.05)*	*0.48 (p = 0.001)*	*0.30 (p = 0.01)*	*0.23 (p = 0.05)*	1.00			
HDL-C	53.5	10.0	0.17	−0.06	−0.10	*−0.21 (p = 0.05)*	−0.11	1.00		
	49.6	7.1	−0.13	*−0.37 (p = 0.001)*	*−0.23 (p = 0.05)*	−0.18	*−0.48 (p = 0.001)*	1.00		
non-HDL-C	129.6	27.1	0.16	*0.29 (p = 0.01)*	*0.28 (p = 0.01)*	*0.34 (p = 0.001)*	*0.89 (p = 0.001)*	*−0.47 (p = 0.001)*	1.00	
	134.9	31.4	*0.22 (p = 0.05)*	*0.49 (p = 0.001)*	*0.32 (p = 0.01)*	*0.22 (p = 0.05)*	*0.95 (p = 0.001)*	*−0.68 (p = 0.001)*	1.00	
TC/HDL	3.5	0.9	−0.03	0.19	*0.22 (p = 0.05)*	*0.32 (p = 0.01)*	*0.57 (p = 0.001)*	*−0.83 (p = 0.001)*	*0.85 (p = 0.001)*	1.00
	3.8	1.0	0.17	*0.47 (p = 0.001)*	*0.31 (p = 0.01)*	*0.22 (p = 0.05)*	*0.80 (p = 0.001)*	*−0.88 (p = 0.001)*	*0.93 (p = 0.001)*	1.00

Note: * first row corresponds to first assessment (r_s_crit (n2015/2017 = 96, *p* = 0.05)_ = 0.201); ** second row corresponds to second assessment (r_s_crit (n2017/2020 = 93, *p* = 0.05)_ = 0.204); italic represents significant Spearman correlation coefficient (significance level in brackets).

**Table 3:** Spearman correlation coefficient matrix between age, BMI, CRP and lipid profile determined in investigated patients and healthy volunteers according to first and second assessment period and type of diet.

Diet Type	Variable	Age	BMI	CRP	TG	TC	HDL-C	Non-HDL-C	TC/HDL
LFD	Age	1.00 *							
		1.00 **							
	BMI	0.23	1.00						
		0.35	1.00						
	CRP	0.01	−0.12	1.00					
		−0.20	−0.28	1.00					
	TG	0.13	0.15	−0.32	1.00				
		−0.26	−0.35	0.26	1.00				
	TC	0.10	−0.09	−0.25	−0.10	1.00			
		0.15	*−0.63*	0.09	0.47	1.00			
	HDL-C	0.11	0.39	0.16	−0.09	−0.12	1.00		
		0.18	0.31	0.30	0.04	−0.39	1.00		
	non-HDL-C	0.11	−0.07	−0.27	−0.02	*0.89*	−0.44	1.00	
		0.00	*−0.60*	0.00	0.31	*0.92*	*−0.68*	1.00	
	TC/HDL	0.07	−0.26	−0.31	0.06	*0.65*	*−0.76*	*0.87*	1.00
		−0.14	−0.51	−0.06	0.28	*0.77*	*−0.85*	*0.94*	1.00
GF–CF	Age	1.00							
		1.00							
	BMI	0.41	1.00						
		−0.30	1.00						
	CRP	−0.02	−0.15	1.00					
		0.62	−0.41	1.00					
	TG	0.21	0.52	−0.15	1.00				
		−0.44	0.41	−0.02	1.00				
	TC^1^	−0.21	−0.04	*−0.64*	−0.22	1.00			
		0.02	*0.84*	−0.16	0.39	1.00			
	HDL-C	−0.04	−0.18	*0.80*	−0.37	−0.46	1.00		
		−0.04	−0.19	−0.09	−0.41	−0.07	1.00		
	non-HDL-C	−0.05	−0.12	*−0.71*	0.07	*0.88*	*−0.69*	1.00	
		0.17	*0.73*	−0.11	0.30	*0.82*	−0.55	1.00	
	TC/HDL	−0.03	−0.15	*−0.71*	0.16	*0.65*	*−0.89*	*0.88*	1.00
		0.01	0.55	−0.05	0.54	0.56	*−0.84*	*0.88*	1.00
RD	Age	1.00							
		1.00							
	BMI	−0.08	1.00						
		−0.05	1.00						
	CRP	0.15	−0.02	1.00					
		0.07	0.09	1.00					
	TG	−0.16	0.18	−0.07	1.00				
		−0.02	*0.45*	−0.16	1.00				
	TC	0.32	0.16	0.15	0.10	1.00			
		*0.54*	*0.54*	0.08	*0.37*	1.00			
	HDL-C	0.01	0.09	0.25	−0.10	0.10	1.00		
		−0.34	*−0.38*	0.09	−0.32	*−0.53*	1.00		
	non-HDL-C	0.29	0.08	0.02	0.14	*0.91*	−0.24	1.00	
		*0.56*	*0.53*	0.04	0.33	*0.97*	*−0.66*	1.00	
	TC/HDL	0.21	0.03	−0.16	0.20	*0.56*	*−0.72*	*0.81*	1.00
		*0.54*	*0.54*	0.04	0.34	*0.90*	*−0.79*	*0.97*	1.00
CG	Age	1.00							
		1.00							
	BMI	*0.55*	1.00						
		0.21	1.00						
	CRP	−0.07	−0.12	1.00					
		−0.15	−0.12	1.00					
	TG	*0.52*	0.25	−0.07	*0.52*				
		0.24	0.04	−0.01	1.00				
	TC	*0.66*	0.17	0.08	0.14	1.00			
		0.10	0.29	−0.26	−0.15	1.00			
	HDL-C	*0.37*	0.22	0.09	*0.38*	*0.49*	1.00		
		−0.06	−0.11	−0.24	−0.16	*0.47*	1.00		
	non-HDL-C	*0.39*	0.14	0.07	−0.28	*0.69*	−0.17	1.00	
		0.13	*0.41*	−0.25	−0.04	*0.82*	−0.01	1.00	
	TC/HDL	−0.13	−0.15	−0.01	*−0.48*	0.04	*−0.80*	*0.68*	1.00
		0.06	*0.36*	−0.03	0.09	0.28	*−0.65*	*0.72*	1.00

Note: * first row corresponds to first assessment (r_s_crit (nLFD = 14, *p* = 0.05)_ = 0.538; r_s_crit (nGF/CF = 10, *p* = 0.05)_ = 0.648; r_s_crit (nRD = 35, *p* = 0.05)_ = 0.335; r_s_crit (nCG = 37, *p* = 0.05)_ = 0.325); ** second row corresponds to second assessment (r_s_crit (nLFD = 14, *p* = 0.05)_ = 0.538; r_s_crit (nGF/CF = 10, *p* = 0.05)_ = 0.648; r_s_crit (nRD = 33, *p* = 0.05)_ = 0.345; r_s_crit (nCG = 36, *p* = 0.05)_ = 0.330); bold italic represents significant Spearman correlation coefficient.

**Table 4:** The differences over time between blood parameters according to the type of diet for ASD patients and healthy volunteers.

Diet Type	Variable	First Period of Assessment (2015/2017)		Second Period of Assessment (2017/2020)		Number of Pairs (Significance of Wilcoxon’s Test)
		Median	IQR	Median	IQR	
LFD	BMI	24.0	3.0	24.5	1.0	*n* = 12 (*p* = 0.239)
	CRP	6.9	9.9	8.7	10.0	*n* = 13 (*p* = 0.422)
	TG	99.3	17.3	117.2	47.2	*n* = 14 (*p* = 0.109)
	TC	175.1	32.9	189.7	18.9	*n* = 14 (*p* = 0.074)
	HDL-C	50.5	14.6	48.7	9.2	*n* = 14 (*p* = 0.638)
	non-HDL-C	123.2	41.4	138.2	24.1	*n* = 14 (*p* = 0.064)
	TC/HDL	3.4	1.2	3.9	1.1	*n* = 14 (*p* = 0.245)
GF–CF	BMI	24.5	2.0	24.5	3.0	*n* = 5 (*p* = 0.686)
	CRP	5.5	6.9	4.5	8.4	*n* = 10 (*p* = 0.959)
	TG	100.1	41.4	90.5	19.7	*n* = 9 (*p* = 0.441)
	TC	184.6	31.6	197.2	9.8	*n* = 10 (*p* = 0.845)
	HDL-C	49.4	5.7	44.6	8.9	*n* = 10 (*p* = 0.241)
	non-HDL-C	129.5	48.3	151.9	13.1	*n* = 10 (*p* = 0.169)
	TC/HDL	3.6	1.6	4.4	0.9	*n* = 10 (*p* = 0.241)
RD	BMI	25.0	3.0	25.0	4.0	*n* = 25 (*p* = 0.353)
	CRP	9.5	14.3	9.5	8.4	*n* = 31 (*p* = 0.717)
	TG *	147.5	70.6	101.3	41.4	*n* = 32 (*p* = 0.001)
	TC	199.7	35.5	198.7	54.8	*n* = 33 (*p* = 0.893)
	HDL-C *	46.9	12.4	44.2	8.6	*n* = 32 (*p* = 0.035)
	non-HDL-C	146.0	38.1	153.6	60.8	*n* = 33 (*p* = 0.851)
	TC/HDL	4.1	1.4	4.4	2.1	*n* = 33 (*p* = 0.195)
CG	BMI	22.0	5.0	24.0	2.9	*n* = 30 (*p* = 0.067)
	CRP *	1.5	2.4	2.6	3.8	*n* = 30 (*p* = 0.049)
	TG *	81.7	27.0	99.2	27.0	*n* = 36 (*p* < 0.001)
	TC *	167.8	15.3	165.6	12.4	*n* = 35 (*p* = 0.044)
	HDL-C *	56.8	16.1	55.9	9.7	*n* = 35 (*p* = 0.016)
	non-HDL-C	111.9	15.4	111.3	17.0	*n* = 35 (*p* = 0.534)
	TC/HDL	2.9	0.6	3.1	0.5	*n* = 35 (*p* = 0.098)

Note: * means an existence of significant difference between given blood parameter in consecutive assessment periods.
